# Serum β-defensin-3 and inflammatory markers for discriminating diabetic foot infection and infection severity: a retrospective study

**DOI:** 10.3389/fendo.2026.1966400

**Published:** 2026-10-14

**Authors:** Yali Ma, Zhao Qu, Qianqian Yao, Huimin Huang, Chengfu Yuan

**Affiliations:** 1 School of Basic Medical Sciences, China Three Gorges University, Yichang, Hubei, China; 2 Clinical Laboratory, Yuan’an People’s Hospital, Yichang, Hubei, China; 3 School of Health Medicine, China Three Gorges University, Yichang, Hubei, China; 4 Department of Burns, Yichang Central People’s Hospital, Yichang, Hubei, China

**Keywords:** diabetic foot infection, diagnostic discrimination, human β-defensin-3, infection severity, inflammatory markers

## Abstract

**Background:**

Human β-defensin-3 (hBD-3) is an epithelial-derived antimicrobial peptide involved in innate immunity and tissue repair, but its clinical association with diabetic foot infection (DFI) remains insufficiently characterized.

**Objective:**

To evaluate the associations of serum hBD-3 with prevalent DFI and infection severity and to assess its discriminatory performance alone and in combination with conventional inflammatory markers.

**Methods:**

This retrospective study included 150 participants: 30 healthy controls, 30 patients with diabetes without foot disease, and 90 patients with diabetic foot disease, including 30 without infection and 60 with DFI. Logistic regression and LASSO penalized regression were used to identify factors associated with prevalent DFI and moderate/severe infection. Restricted cubic spline analyses explored nonlinear associations. Discriminatory performance was evaluated using receiver operating characteristic curves, and internal validation was performed with 1,000 bootstrap resamples.

**Results:**

Serum hBD-3 was higher in patients with DFI than in those with non-infected diabetic foot disease [108.9 (86.3, 141.5) vs. 69.8 (56.1, 84.7) pg/mL, *P* < 0.001] and increased with infection severity. Higher hBD-3, CRP, PCT, and HbA1c and lower albumin were independently associated with prevalent DFI. The final prevalent-DFI model included HbA1c, albumin, hBD-3, CRP, and PCT and achieved an apparent AUC of 0.928, with an optimism-corrected AUC of 0.895. The moderate/severe DFI model included hBD-3, CRP, and PCT and achieved an apparent AUC of 0.935 and an optimism-corrected AUC of 0.917. RCS analyses suggested nonlinear associations between hBD-3 and both prevalent DFI and infection severity.

**Conclusion:**

Serum hBD-3 was associated with the presence and severity of DFI and provided complementary discriminatory information beyond conventional inflammatory markers. The combined models showed promising performance but remain exploratory and require prospective multicenter external validation before clinical application.

## 1 Introduction

Diabetic foot disease is a common and serious complication of diabetes. Its occurrence is associated with multiple factors, including peripheral neuropathy, peripheral arterial disease, persistent hyperglycemia, and impaired local tissue repair. After disruption of the skin barrier of the foot, local microbial colonization and invasion may further lead to diabetic foot infection (DFI), which can progress from superficial soft-tissue infection to deep-tissue infection and even diabetic foot osteomyelitis. DFI can delay wound healing and prolong hospitalization and is an important reason for surgical debridement or even amputation. Therefore, accurate identification of infection and early assessment of its severity are important components of diabetic foot management. The 2023 International Working Group on the Diabetic Foot/Infectious Diseases Society of America (IWGDF/IDSA) guideline states that DFI is diagnosed mainly on the basis of local or systemic manifestations of infection and recommends grading infection as mild, moderate, or severe according to the IWGDF/IDSA classification to guide treatment (1, 2). Laboratory inflammatory markers provide convenient objective information for the auxiliary identification and severity assessment of DFI. White blood cell count (WBC), C-reactive protein (CRP), erythrocyte sedimentation rate (ESR), procalcitonin (PCT), and neutrophil-to-lymphocyte ratio (NLR) reflect the inflammatory response from different perspectives. When the clinical diagnosis of DFI is uncertain, CRP, ESR, or PCT can be used as adjunctive indicators, although the sensitivity and specificity of these markers when used individually remain limited. Zhang et al. (3) analyzed 301 patients with moderate or severe DFI and found that CRP was independently associated with infection severity, with a nonlinear dose-response relationship, suggesting that the relationship between inflammatory markers and DFI progression is not simply linear. Zhao et al. (4) reported that perioperative NLR was associated with wound healing in patients with Wagner grade 3–4 diabetic foot ulcers, indicating that blood cell-derived inflammatory indices may also have potential value in the assessment of diabetic foot disease. However, DFI development involves microbial invasion, disruption of the skin barrier, abnormal host immune responses, and impaired tissue repair. Conventional markers reflecting systemic inflammation alone may therefore be insufficient to characterize this complex pathological process. Human β-defensin-3 (hBD-3) is an important member of the β-defensin family and an epithelial-derived host defense peptide closely involved in innate immunity and antimicrobial defense of the skin and mucosa. Recent studies have shown that, in addition to direct antimicrobial activity, antimicrobial peptides may participate in inflammatory regulation, angiogenesis, keratinocyte migration, and tissue repair and may therefore have biological relevance to diabetic wound healing (5). Tsuji et al. (6) showed that hBD-3 production in human keratinocytes is regulated by CREB- and NRF2-related signaling pathways, providing new experimental evidence for its role in innate epithelial defense. Diabetes-related foot wounds are chronically exposed to hyperglycemia, ischemia and hypoxia, oxidative stress, persistent inflammation, skin-barrier injury, and microbial invasion, all of which may alter hBD-3 expression and host defense responses. However, clinical evidence regarding differences in serum hBD-3 among healthy individuals, patients with diabetes alone, and patients with diabetic foot disease, as well as its association with the presence and severity of DFI, remains limited. Whether hBD-3 provides discriminatory information beyond conventional inflammatory markers is also unclear. It is important to distinguish diagnostic or discriminatory biomarkers from prognostic biomarkers. Diagnostic biomarkers are used to identify or distinguish an existing disease or clinical state at the time of assessment, whereas prognostic biomarkers are intended to estimate the likelihood of future clinical outcomes or disease progression. In the present study, serum hBD-3 and inflammatory markers were measured contemporaneously with the clinical assessment of infection status. Therefore, the present analysis focuses on their associations with prevalent DFI and their ability to discriminate between clinically defined infection states, rather than on prediction of future DFI development or subsequent prognosis. Accordingly, this study compared serum hBD-3 levels among healthy controls, patients with diabetes alone, and patients with diabetic foot disease; further stratified patients according to infection status and severity; and examined the associations of hBD-3 with conventional inflammatory markers and prevalent DFI. Logistic regression, restricted cubic spline analyses, and receiver operating characteristic curves were used to characterize these associations and to evaluate the discriminatory performance of hBD-3 alone and in combination with inflammatory markers for distinguishing DFI from non-infected diabetic foot disease and moderate/severe from mild infection.

## 2 Materials and methods

### 2.1 Study population

Between January 2025 and January 2026, 247 potentially eligible individuals were retrospectively screened through the electronic medical record system, including 125 patients with diabetic foot disease, 62 patients with diabetes without foot disease, and 60 healthy individuals undergoing physical examination. Among the 125 patients with diabetic foot disease, 35 were excluded because of active infection at other sites (n=6), systemic antimicrobial therapy for >72 h before index blood sampling (n=8), severe comorbidities or other predefined exclusion criteria (n=5), absence of an eligible stored serum sample (n=9), or missing key clinical variables required for the primary analyses (n=7). All 90 eligible patients with diabetic foot disease were consecutively included, comprising 30 patients with non-infected diabetic foot disease and 60 patients with DFI. According to the IWGDF/IDSA criteria, the 60 patients with DFI were classified as mild (n=18), moderate (n=29), or severe (n=13). Among the 62 potentially eligible patients with diabetes without foot disease, 12 were excluded because of active infection (n=3), absence of an eligible stored serum sample (n=5), or missing key clinical data (n=4), leaving 50 eligible patients. Similarly, among 60 potentially eligible healthy controls, 10 were excluded because of infectious or inflammatory conditions (n=4), absence of an eligible stored serum sample (n=3), or missing key data (n=3), leaving 50 eligible individuals. From each of these two eligible reference populations, 30 participants were selected using computer-generated simple random sampling to provide balanced reference groups of the same size as the non-infected diabetic foot disease group (n=30). These reference groups were included primarily for biological comparisons and were not used for development of the primary DFI discrimination model, whereas the primary clinical analyses were based on the complete cohort of 90 patients with diabetic foot disease. No individual matching was performed. A total of 150 participants were therefore included in the final analysis (**Figure 1**). Terminology was standardized throughout the manuscript. “Diabetic foot disease” was used as the overarching term for diabetes-related foot pathology in the study population. “Non-infected diabetic foot disease” referred specifically to patients with an active diabetic foot ulcer without clinical evidence of infection, whereas “diabetic foot infection (DFI)” was reserved for patients meeting the IWGDF/IDSA diagnostic criteria for infection. The term “diabetic foot ulcer (DFU)” was used only when referring specifically to ulcerative lesions or when describing studies that enrolled patients with DFU. For the present analysis, the non-infected diabetic foot disease group consisted exclusively of patients with active diabetic foot ulcers who showed no clinical evidence of infection according to the IWGDF/IDSA criteria. All 30 patients in this group had an active foot ulcer but did not meet the clinical diagnostic criteria for DFI at the index assessment. Patients with isolated Charcot neuroarthropathy without ulceration, pre-ulcerative lesions alone, or other non-ulcerative diabetic foot conditions were not included in this comparison group. Ischemic or neuroischemic ulcers and dry gangrene could be present provided that there were no clinical signs of active infection. DFI was diagnosed when at least two local manifestations of inflammation or purulent discharge were present, in accordance with the IWGDF/IDSA criteria. According to the International Working Group on the Diabetic Foot/Infectious Diseases Society of America (IWGDF/IDSA) criteria (1), the infection group was classified as mild infection (n=18), moderate infection (n=29), or severe infection (n=13). According to the IWGDF/IDSA classification, severe DFI (grade 4) was defined as a foot infection accompanied by at least two systemic inflammatory response syndrome (SIRS) criteria, including body temperature >38 °C or <36 °C, heart rate >90 beats/min, respiratory rate >20 breaths/min or PaCO_2_ <32 mmHg, or a white blood cell count >12,000/mm^3^, <4,000/mm^3^, or >10% immature band forms. Severe DFI according to the IWGDF/IDSA classification was distinguished from sepsis as defined by the Sepsis-3 criteria. In the present study, sepsis was defined as infection-associated life-threatening acute organ dysfunction, operationalized as an acute increase in the Sequential Organ Failure Assessment (SOFA) score of ≥2 points. Thus, fulfillment of the SIRS criteria for severe DFI alone was not considered equivalent to sepsis. Ulcer severity was also assessed using the Wagner classification (7). Infection grade was independently determined by two physicians experienced in the diagnosis and treatment of diabetic foot disease. Disagreements were reviewed by a third senior physician.

**Figure 1:**
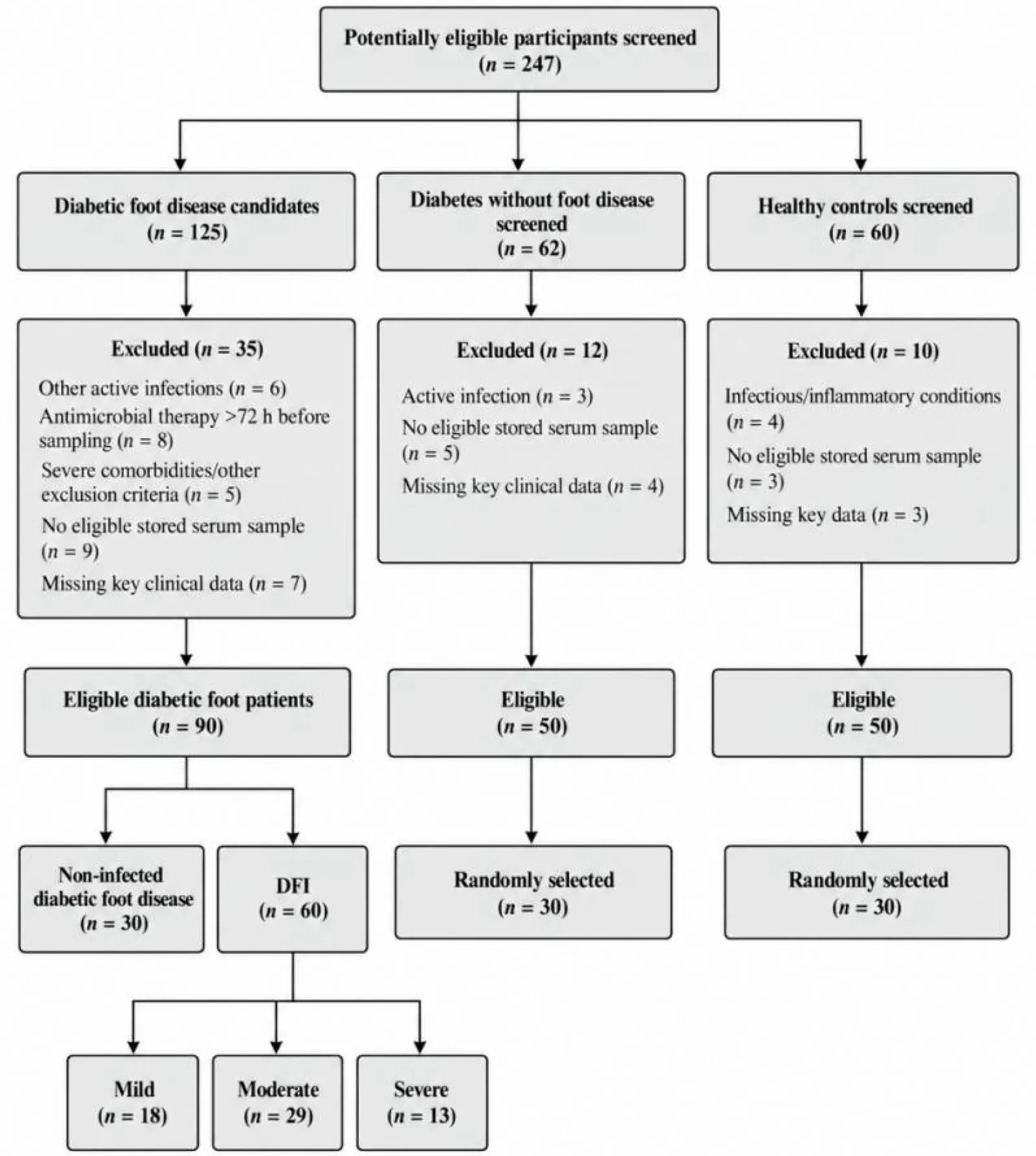
Summarizes the screening and allocation of study participants. A total of 247 potentially eligible individuals were screened, and 150 participants were ultimately included, comprising 30 healthy controls, 30 patients with diabetes without foot disease, 30 patients with non-infected diabetic foot disease, and 60 patients with DFI. The 60 DFI cases were further classified as mild (n=18), moderate (n=29), and severe (n=13).

Inclusion criteria: (1) age 18–74 years; (2) complete clinical data and availability of stored serum obtained at the same time as the clinical assessment; (3) patients with diabetes met the diagnostic criteria for type 2 diabetes mellitus (8); (4) patients in the non-infected diabetic foot disease group had an active diabetic foot ulcer but showed no clinical evidence of infection and did not meet the IWGDF/IDSA diagnostic criteria for DFI at the index assessment; (5) patients in the diabetes mellitus alone group had no diabetic foot disease, foot ulcer, or active infection; and (6) healthy controls had no diabetes, diabetic foot disease, acute or chronic infection, immune disease, or clearly defined inflammatory condition.

Exclusion criteria: (1) active infection of the lungs, urinary tract, abdominal cavity, or other sites; (2) severe cardiac, hepatic, or renal insufficiency or decompensated disease; (3) malignant tumors, hematologic diseases, autoimmune diseases, or immunodeficiency; (4) use of glucocorticoids, immunosuppressants, or biologic agents within the previous 3 months; (5) Patients who had received systemic antimicrobial therapy for >72 h before the index blood sampling were excluded. Patients with ≤72 h of prior antimicrobial exposure remained eligible, and the duration of antimicrobial treatment before blood sampling was recorded; (6) recent severe trauma, burns, surgery, or other wounds unrelated to diabetic foot disease; (7) sepsis meeting the Sepsis-3 criteria, defined as infection-associated acute organ dysfunction with an acute increase in SOFA score of ≥2 points, septic shock, disseminated intravascular coagulation, or other critical systemic complications requiring intensive care; (8) pregnancy or lactation. The study protocol was reviewed and approved by the Ethics Committee of Yuan’an County People’s Hospital (approval No. 2026YAX213-01) on April 1, 2026. Because this was a retrospective study involving the analysis of existing clinical data and previously stored serum samples, with no additional intervention to participants, the requirement for written informed consent was waived by the Ethics Committee. All data were de-identified before analysis. The secondary use of previously stored serum samples for the measurement of serum hBD-3 and related laboratory analyses was specifically covered by this ethical approval. The study was reported in accordance with the STROBE recommendations for observational studies, with relevant STARD principles additionally considered for reporting diagnostic discrimination analyses.

### 2.2 Collection of clinical data

Age, sex, body mass index, smoking history, duration of diabetes, glucose-lowering regimen, hypertension, coronary heart disease, chronic kidney disease, peripheral arterial disease, peripheral neuropathy, fasting blood glucose, glycated hemoglobin, albumin, creatinine, blood urea nitrogen, and estimated glomerular filtration rate were extracted from the electronic medical record, laboratory information, and imaging systems. For all patients with diabetic foot disease, wound-related characteristics were systematically extracted, including ulcer duration, ulcer area, ulcer depth, Wagner grade, anatomical location, peripheral arterial disease, peripheral neuropathy, osteomyelitis, gangrene, purulent discharge, exudation, necrosis, odor, and antimicrobial exposure before the index clinical assessment. Ulcer depth was categorized according to the deepest tissue involved, and anatomical location was classified as forefoot, midfoot, or hindfoot. Previous antimicrobial exposure was defined as receipt of systemic antibacterial therapy before the index blood sampling. Patients who had received systemic antimicrobial therapy for >72 h before sampling were excluded, whereas those with ≤72 h of prior exposure remained eligible. For exposed patients, the antimicrobial agent and duration of treatment before blood sampling were recorded when available. Index laboratory measurements were obtained at the first clinical assessment or within 24 h of admission and before surgical debridement. Whenever possible, blood samples were collected before initiation of systemic antimicrobial therapy. These wound-related and treatment-related characteristics were compared between patients with non-infected diabetic foot disease and those with DFI. Among patients with DFI, prior antimicrobial exposure and treatment duration before blood sampling were further compared according to infection severity. For patients with DFI, results of routine microbiological cultures obtained during clinical care were extracted from the medical records when available. Culture positivity, isolated microorganisms, broad microbiological categories, polymicrobial infection, and osteomyelitis status were summarized. Because microbiological culture data were unavailable for some patients and the resulting subgroups were small, comparisons of serum hBD-3 according to osteomyelitis status and broad microbiological categories were prespecified as exploratory analyses.

### 2.3 Measurement of serum hBD-3 and inflammatory markers

Serum samples were centrifuged after collection, aliquoted, and stored at −80 °C until analysis. Based on the sample-collection period from January 2025 to January 2026, the maximum storage duration before hBD-3 measurement was approximately 12 months. Samples underwent no more than one freeze–thaw cycle before analysis. Serum human β-defensin-3 (hBD-3) concentrations were measured using a Human BD-3 SimpleStep ELISA Kit (Abcam Ltd., Cambridge, UK; catalogue no. ab272471), a quantitative colorimetric sandwich ELISA. According to the manufacturer’s reported analytical performance, the nominal calibration range was 31.25–2,000 pg/mL, and the assay sensitivity, corresponding to the reported lower limit of detection (LOD), was 14.3 pg/mL. The reported intra-assay and inter-assay coefficients of variation for serum samples were 2.4% and 3.8%, respectively. Sample dilution was selected according to the expected concentration and observed assay signal rather than applying a single fixed dilution factor to all samples. Samples generating signals above the upper limit of the calibration curve were further diluted with the supplied Sample Diluent NS and reassayed, and the reported concentrations were corrected for the corresponding dilution factors. Samples generating signals below the lowest calibrator were reassayed after reducing the dilution factor to obtain measurements within the usable calibration range. All samples were measured in duplicate, and the mean of the duplicate measurements was used for statistical analysis. No sample had a final hBD-3 concentration below the reported LOD after repeat measurement or appropriate adjustment of the dilution factor; therefore, no substitution or imputation for values below the detection limit was required. All 150 serum samples were analyzed during the same analytical campaign using the same reagent lot and standardized operating conditions. Because the number of samples required the use of multiple ELISA plates, samples from the different clinical groups were randomly distributed across plates rather than analyzed sequentially according to clinical group. Laboratory personnel performing the hBD-3 assays were blinded to participants’ clinical group and IWGDF/IDSA infection-severity classification. To minimize potential batch- and plate-related effects, the same reagent lot, incubation conditions, washing procedures, plate-reader settings, and quality-control criteria were maintained across all ELISA plates. Duplicate measurements and internal quality-control procedures were used throughout the assay. The random distribution of samples from different clinical groups across plates was also used to reduce systematic plate-related bias. Nevertheless, residual inter-plate variation could not be completely excluded and was therefore considered when interpreting the hBD-3 results. WBC, neutrophil count, and lymphocyte count were measured using an automated hematology analyzer, and the neutrophil-to-lymphocyte ratio (NLR) was calculated. CRP, PCT, albumin, creatinine, blood urea nitrogen, fasting blood glucose, and glycated hemoglobin were measured according to standard laboratory procedures. ESR was measured using the Westergren method or an automated erythrocyte sedimentation analyzer.

### 2.4 Statistical analysis

Statistical analyses were performed using SPSS version 26.0 and R version 4.4.1. Continuous variables were assessed for normality using the Shapiro–Wilk test. Normally distributed continuous variables were expressed as mean ± standard deviation and compared using the independent-samples t test or one-way analysis of variance, as appropriate. Non-normally distributed variables were expressed as median (P25, P75) and compared using the Mann–Whitney U test or Kruskal–Wallis H test. Categorical variables were expressed as n (%) and compared using the χ^2^ test or Fisher’s exact test, as appropriate. Bonferroni correction was applied for multiple pairwise comparisons when required. Spearman rank correlation analysis was used to assess associations between serum hBD-3 and infection severity or other clinical indicators. Prevalent DFI and moderate/severe DFI were analyzed as binary outcomes. Because biomarker measurements and clinical assessment of infection status were performed contemporaneously, the analyses were interpreted as association and diagnostic-discrimination analyses rather than prospective risk-prediction analyses. Variables with *P* < 0.10 in univariate logistic regression, together with clinically relevant variables, were considered candidate variables for multivariable model development. This screening threshold was used to avoid premature exclusion of potentially informative variables and was not used as the sole basis for final model specification. Sample-size adequacy for model development was assessed retrospectively because this retrospective study included all eligible participants available during the predefined study period and no formal *a priori* sample-size calculation for multivariable model development had been performed. For the prevalent DFI analysis, 60 DFI cases and 30 non-infected diabetic foot disease cases were available. Ten variables were initially considered, corresponding to an initial event-per-variable ratio of approximately 6.0 when DFI cases were used as the event count. After penalized variable selection, five variables were retained in the final model. For the moderate/severe DFI analysis, 42 moderate/severe cases and 18 mild cases were available; six candidate biomarkers were initially considered and three were retained in the final model. Although the number of retained predictors was reduced by penalized selection, the relatively small sample size, particularly the small number of patients in the smaller outcome category, continued to impose a risk of unstable coefficient estimates and model overfitting. Therefore, all model-development analyses were considered exploratory, and bootstrap internal validation was used to quantify optimism. Given the limited sample size, least absolute shrinkage and selection operator (LASSO) penalized logistic regression was used for variable selection. Stratified 10-fold cross-validation was performed, and the λ1se value was selected to obtain more parsimonious models. Variables with non-zero coefficients at λ1se were subsequently entered into multivariable logistic regression models. Associations were reported as odds ratios (ORs) with 95% confidence intervals (95% CIs). Variance inflation factors (VIFs) were calculated to assess multicollinearity. hBD-3 and inflammatory biomarkers were analyzed as continuous variables using the prespecified measurement increments reported in the corresponding regression tables. To address potential confounding by wound severity, a sensitivity analysis of the association between serum hBD-3 and prevalent DFI was additionally adjusted for ulcer duration, ulcer area, Wagner grade, and peripheral arterial disease. Exploratory restricted cubic spline (RCS) analyses were performed to examine potential nonlinear associations. Three knots were placed at the 10th, 50th, and 90th percentiles of each biomarker distribution. Given the limited sample size, this parsimonious three-knot specification was selected to balance model flexibility against the risk of unstable estimates and overfitting. The median biomarker value, corresponding to the 50th-percentile knot, was used as the reference value. For prevalent DFI, the hBD-3 spline model was adjusted for HbA1c, albumin, CRP, and PCT, whereas the CRP spline model was adjusted for HbA1c, albumin, hBD-3, and PCT. For moderate/severe DFI, the hBD-3 spline model was adjusted for CRP and PCT. Overall associations and tests for nonlinearity were reported separately. Rug plots were added to indicate the underlying distribution of individual observations. Because observations were sparse at the extremes of the biomarker distributions, all RCS analyses were regarded as exploratory. Receiver operating characteristic (ROC) curves were used to evaluate the ability of individual biomarkers and the final combined models to discriminate prevalent DFI from non-infected diabetic foot disease and moderate/severe DFI from mild DFI. Areas under the ROC curve (AUCs) with 95% CIs, sensitivity, and specificity were reported. AUCs of correlated ROC curves were compared using the DeLong test. Predicted probabilities for the combined models were calculated from the corresponding final logistic regression equations as *P*=exp(LP)/[1+exp(LP)]. ROC-derived cutoff values were considered sample-specific exploratory thresholds and were not interpreted as universally applicable clinical cutoffs. Internal validation was performed using 1,000 bootstrap resamples. In each bootstrap resample, the complete model-development procedure, including LASSO tuning and variable selection followed by refitting of the multivariable logistic regression model, was repeated. Model performance was evaluated in both the bootstrap sample and the original dataset to estimate optimism. Apparent and optimism-corrected AUCs, calibration intercepts, calibration slopes, and Brier scores were calculated, and bootstrap-corrected calibration curves were generated. These internally validated performance estimates were interpreted cautiously because no independent external validation cohort was available. Missing data were assessed before statistical analysis. Fourteen potentially eligible individuals were excluded during screening because of missing key clinical variables, including 7 patients with diabetic foot disease, 4 patients with diabetes without foot disease, and 3 healthy controls. Among the 150 participants included in the final dataset, no values were missing for variables used in the primary regression, RCS, or ROC analyses; therefore, no statistical imputation was performed and complete-case analyses were used. All statistical tests were two-sided, and *P* < 0.05 was considered statistically significant.

## 3 Results

### 3.1 Comparison of clinical characteristics

A total of 150 participants were included, comprising 30 healthy controls, 30 patients with diabetes mellitus alone, 30 patients with non-infected diabetic foot disease, and 60 patients with DFI. Among the three diabetes-related groups, the duration of diabetes differed significantly (*P* = 0.001), whereas the glucose-lowering regimen did not differ significantly (*P* = 0.201). Peripheral arterial disease, peripheral neuropathy, fasting blood glucose, HbA1c, and albumin differed significantly among the four groups (all *P* < 0.001), Age, sex, BMI, smoking history, hypertension, coronary heart disease, chronic kidney disease, creatinine, blood urea nitrogen, and eGFR showed no statistically significant between-group differences (**Table 1**).

**Table 1:** Comparison of clinical characteristics among the four groups.

Indicator	Healthy control group (n=30)	Diabetes mellitus group (n=30)	Non-infected diabetic foot disease group (n=30)	DFI group (n=60)	Statistic	*P* value
Age (mean ± SD, years)	57.9 ± 8.6	59.0 ± 8.9	60.1 ± 9.0	60.8 ± 9.3	*F* = 0.774	0.510
Sex [n (%)]					*χ^2^* = 0.208	0.976
Male	17 (56.7)	18 (60.0)	18 (60.0)	37 (61.7)		
Female	13 (43.3)	12 (40.0)	12 (40.0)	23 (38.3)		
BMI (kg/m^2^)	23.8 ± 2.5	24.1 ± 2.6	24.4 ± 2.7	24.7 ± 2.8	*F* = 0.850	0.469
Smoking history [n (%)]	7 (23.3)	9 (30.0)	10 (33.3)	23 (38.3)	*χ^2^* = 2.167	0.538
Duration of diabetes (mean ± SD, years)		7.4 ± 3.1	9.6 ± 4.0	10.9 ± 4.5	*F* = 7.410	0.001
Glucose-lowering regimen					*χ^2^* = 5.976	0.201
Oral antidiabetic drugs [n (%)]		18 (60.0)	13 (43.3)	20 (33.3)		
Insulin [n (%)]		5 (16.7)	6 (20.0)	14 (23.3)		
Oral drugs + insulin [n (%)]		7 (23.3)	11 (36.7)	26 (43.3)		
Hypertension [n (%)]	8 (26.7)	11 (36.7)	12 (40.0)	27 (45.0)	*χ^2^* = 2.909	0.406
Coronary heart disease [n (%)]	3 (10.0)	5 (16.7)	6 (20.0)	13 (21.7)	*χ^2^* = 1.965	0.580
Chronic kidney disease [n (%)]	2 (6.7)	3 (10.0)	4 (13.3)	9 (15.0)	Fisher	0.756
Peripheral arterial disease [n (%)]	1 (3.3)	3 (10.0)	9 (30.0)	25 (41.7)	*χ^2^* = 20.213	<0.001
Peripheral neuropathy [n (%)]	0 (0.0)	7 (23.3)	18 (60.0)	42 (70.0)	*χ^2^* = 48.175	<0.001
Fasting blood glucose (mmol/L)	5.10 ± 0.48	7.43 ± 1.24	7.96 ± 1.46	8.74 ± 1.72	*F* = 45.721	<0.001
HbA1c (mean ± SD, %)	5.38 ± 0.39	7.27 ± 0.90	7.83 ± 1.08	8.59 ± 1.25	*F* = 66.659	<0.001
Albumin (mean ± SD, g/L)	42.8 ± 3.1	41.1 ± 3.3	39.0 ± 3.6	36.2 ± 4.0	*F* = 26.321	<0.001
Creatinine (mean ± SD, μmol/L)	69.2 ± 14.0	72.4 ± 15.8	75.1 ± 17.5	77.5 ± 18.8	*F* = 1.736	0.162
Blood urea nitrogen (mean ± SD, mmol/L)	5.01 ± 1.05	5.27 ± 1.14	5.48 ± 1.25	5.69 ± 1.34	*F* = 2.245	0.086
eGFR [mean ± SD, mL/(min·1.73 m^2^)]	93.3 ± 13.6	90.4 ± 14.7	87.0 ± 16.0	84.6 ± 17.4	*F* = 2.293	0.081

Duration of diabetes and glucose-lowering regimen were compared only among the three diabetes-related groups. BMI, body mass index; HbA1c, glycated hemoglobin; eGFR, estimated glomerular filtration rate.

### 3.2 Comparison of serum hBD-3 and inflammatory markers according to disease status

Serum hBD-3 levels in the healthy control group, diabetes mellitus group, and diabetic foot disease group were 36.8 (33.7, 40.5), 49.7 (40.5, 61.2), and 93.6 (68.4, 128.7) pg/mL, respectively, with a statistically significant difference among groups (H = 72.416, *P* < 0.001). WBC, CRP, ESR, PCT, and NLR were higher in the diabetic foot disease group than in the healthy control and diabetes mellitus groups (all *P* < 0.001) (**Table 2**).

**Table 2:** Comparison of serum hBD-3 and inflammatory markers among the three groups.

Indicator	Healthy control group (n=30)	Diabetes mellitus group (n=30)	Diabetic foot disease group (n=90)	Statistic	*P* value
hBD-3 [pg/mL, M (P25, P75)]	36.8 (33.7, 40.5)	49.7 (40.5, 61.2)	93.6 (68.4, 128.7)	*H* = 72.416	<0.001
WBC (×10^9^/L)	5.82 ± 1.12	6.18 ± 1.27	8.59 ± 2.28	*F* = 33.525	<0.001
CRP [mg/L, M (P25, P75)]	1.9 (1.1, 2.9)	3.8 (2.2, 5.8)	27.5 (11.2, 55.7)	*H* = 77.528	<0.001
ESR [mm/h, M (P25, P75)]	8 (5, 12)	14 (9, 21)	42 (27, 62)	*H* = 70.316	<0.001
PCT [ng/mL, M (P25, P75)]	0.03 (0.02, 0.05)	0.04 (0.03, 0.06)	0.15 (0.07, 0.35)	*H* = 63.482	<0.001
NLR [M (P25, P75)]	1.71 (1.39, 2.07)	2.01 (1.61, 2.50)	3.55 (2.52, 4.89)	*H* = 56.744	<0.001

### 3.3 Comparison of wound-related clinical characteristics between non-infected diabetic foot disease and DFI

Among the 90 patients with diabetic foot disease, 30 had active diabetic foot ulcers without clinical evidence of infection and were classified as the non-infected diabetic foot disease group, whereas 60 met the IWGDF/IDSA diagnostic criteria for DFI. A detailed wound-related comparison between these two clinically relevant groups is presented in **Table 3**. Compared with the non-infected diabetic foot disease group, patients with DFI had a longer ulcer duration [66.1 (37.9, 110.6) vs. 32.7 (24.6, 42.7) d, *P* < 0.001] and a larger ulcer area [5.0 (3.2, 9.1) vs. 2.2 (1.9, 3.9) cm^2^, *P* < 0.001]. Ulcer depth and Wagner grade distributions also differed significantly between the groups (both *P* < 0.001), with deeper ulcers and higher Wagner grades occurring more frequently in patients with DFI. Osteomyelitis was also more common in the DFI group than in the non-infected diabetic foot disease group (26.7% vs. 0.0%, *P* = 0.001). In contrast, peripheral arterial disease (P = 0.282), peripheral neuropathy (*P* = 0.343), gangrene (*P* = 0.324), anatomical location (*P* = 0.832), and previous systemic antimicrobial exposure (*P* = 0.201) did not differ significantly between the two groups (**Table 3**).

**Table 3:** Comparison of wound-related clinical characteristics between non-infected diabetic foot disease and DFI.

Variable	Non-infected diabetic foot disease group (n=30)	DFI group (n=60)	Statistic	*P* value
Ulcer duration, d, M (P25, P75)	32.7 (24.6, 42.7)	66.1 (37.9, 110.6)	*Z*=−4.194	<0.001
Ulcer area, cm^2^, M (P25, P75)	2.2 (1.9, 3.9)	5.0 (3.2, 9.1)	*Z*=−3.535	<0.001
Ulcer depth, n (%)			*χ^2^* = 22.093	<0.001
Skin/subcutaneous tissue	22 (73.3)	15 (25.0)		
Tendon/fascia/muscle	8 (26.7)	27 (45.0)		
Bone/joint involvement	0 (0.0)	18 (30.0)		
Wagner grade, n (%)			*χ^2^* = 32.629	<0.001
Grade 1	17 (56.7)	5 (8.3)		
Grade 2	11 (36.7)	21 (35.0)		
Grade 3	0 (0.0)	24 (40.0)		
Grade 4	2 (6.7)	10 (16.7)		
Peripheral arterial disease, n (%)	9 (30.0)	25 (41.7)	*χ^2^* = 1.158	0.282
Peripheral neuropathy, n (%)	18 (60.0)	42 (70.0)	*χ^2^* = 0.900	0.343
Osteomyelitis, n (%)	0 (0.0)	16 (26.7)	Fisher	0.001
Gangrene, n (%)	2 (6.7)	10 (16.7)	Fisher	0.324
Anatomical location, n (%)			*χ*^2^=0.369	0.832
Forefoot	19 (63.3)	34 (56.7)		
Midfoot	5 (16.7)	12 (20.0)		
Hindfoot	6 (20.0)	14 (23.3)		
Previous systemic antimicrobial exposure, n (%)	4 (13.3)	15 (25.0)	χ^2^=1.635	0.201

DFI, diabetic foot infection. Continuous variables are presented as M (P25, P75) and were compared using the Mann–Whitney U test. Categorical variables are presented as n (%) and were compared using the χ^2^ test or Fisher’s exact test, as appropriate. Wagner grade was assessed at the index clinical evaluation. Previous antimicrobial exposure refers to systemic antimicrobial treatment before the index assessment.

### 3.4 Prior antimicrobial exposure before blood sampling

All 13 patients classified as having severe DFI fulfilled the SIRS-based criteria for IWGDF/IDSA grade 4 infection. At the index assessment, none met the Sepsis-3 criteria for infection-associated acute organ dysfunction (acute increase in SOFA score ≥2), septic shock, or disseminated intravascular coagulation. These patients were therefore retained in the severe DFI group rather than excluded as patients with sepsis. Among the 60 patients with DFI, 15 (25.0%) had received systemic antimicrobial therapy before the index blood sampling. The median duration of antimicrobial exposure before sampling was 24 h (P25, P75: 18, 33 h), and no included patient had received systemic antimicrobial therapy for >72 h. Prior antimicrobial exposure was documented in 3 of 18 patients with mild DFI (16.7%), 7 of 29 with moderate DFI (24.1%), and 5 of 13 with severe DFI (38.5%). The proportions did not differ significantly across infection-severity groups (Fisher-Freeman-Halton exact test, *P* = 0.424). Among patients with prior antimicrobial exposure, the median treatment durations were 18 (15, 21), 24 (21, 33), and 30 (24, 36) h in the mild, moderate, and severe groups, respectively, with no statistically significant between-group difference (*H* = 2.950, *P* = 0.229) (**Table 4**).

**Table 4:** Antimicrobial exposure before blood sampling according to DFI severity.

Variable	Mild (n=18)	Moderate (n=29)	Severe (n=13)	Total (n=60)	Statistic	*P*
Prior antimicrobial exposure, n (%)	3 (16.7)	7 (24.1)	5 (38.5)	15 (25.0)	Fisher-Freeman-Halton	0.424
Duration before sampling, h, M (P25, P75)	18 (15, 21)	24 (21, 33)	30 (24, 36)	24 (18, 33)	*H* = 2.950	0.229

Duration was calculated only among patients who had received antimicrobial therapy before blood sampling. Patients receiving systemic antimicrobial therapy for >72 h before sampling were excluded.

### 3.5 Comparison of serum hBD-3 and inflammatory markers between non-infected and DFI patients

Among the 90 patients with diabetic foot disease, 30 had non-infected diabetic foot disease and 60 had DFI. Serum hBD-3 was significantly higher in patients with DFI than in those with non-infected diabetic foot disease [108.9 (86.3, 141.5) vs. 69.8 (56.1, 84.7) pg/mL, *P* < 0.001]. WBC, CRP, ESR, PCT, and NLR were also significantly higher in the DFI group (all *P* < 0.001) (**Table 5**).

**Table 5:** Comparison of laboratory indicators between the non-infected diabetic foot disease group and the DFI group.

Indicator	Non-infected diabetic foot disease group (n=30)	DFI group (n=60)	Statistic	*P* value
hBD-3 (pg/mL)	69.8 (56.1, 84.7)	108.9 (86.3, 141.5)	*Z* = -5.126	<0.001
WBC (×10^9^/L)	7.04 ± 1.52	9.42 ± 2.11	*t* = -5.187	<0.001
CRP (mg/L)	9.8 (5.9, 15.4)	39.1 (19.7, 70.0)	*Z* = -5.068	<0.001
ESR (mm/h)	27 (18, 38)	52 (36, 71)	*Z* = -4.315	<0.001
PCT (ng/mL)	0.07 (0.04, 0.11)	0.23 (0.11, 0.49)	*Z* = -4.698	<0.001
NLR	2.67 (2.05, 3.31)	4.24 (3.05, 5.79)	*Z* = -4.106	<0.001

### 3.6 Microbiological characteristics, osteomyelitis, and exploratory hBD-3 comparisons

Among the 60 patients with DFI, microbiological cultures were available for 56 (93.3%), of whom 44 (78.6%) were culture positive. A total of 57 clinically relevant microorganisms were isolated. The most frequently isolated organisms were Staphylococcus aureus (28.1%), Pseudomonas aeruginosa (19.3%), and Escherichia coli (15.8%). Among the 44 culture-positive patients, 17 (38.6%) had Gram-positive organisms only, 14 (31.8%) had Gram-negative organisms only, and 13 (29.5%) had polymicrobial infection. Osteomyelitis was present in 16 of 60 patients with DFI (26.7%) (**Table 6**). Exploratory analyses showed that serum hBD-3 concentrations were higher in patients with osteomyelitis than in those without osteomyelitis [150.0 (137.0, 163.5) vs. 119.0 (97.5, 140.5) pg/mL, *U* = 561.000, *P* < 0.001]. Serum hBD-3 levels also differed significantly among patients with Gram-positive organisms only, Gram-negative organisms only, and polymicrobial infection [112.0 (100.0, 128.0), 121.5 (107.3, 136.5), and 146.0 (134.0, 160.0) pg/mL, respectively; *H* = 12.906, *P* = 0.002]. After Bonferroni correction, hBD-3 levels were higher in the polymicrobial group than in the Gram-positive-only (*P* = 0.002) and Gram-negative-only (*P* = 0.035) groups, whereas the difference between the Gram-positive-only and Gram-negative-only groups was not statistically significant (*P* = 0.933) (**Table 7**). Given the limited subgroup sample sizes, these analyses were considered exploratory.

**Table 6:** Microbiological characteristics and distribution of isolated microorganisms in patients with DFI.

Characteristic	n (%)/isolates (%)
Microbiological culture performed	56/60 (93.3)
Culture positive	44/56 (78.6)
Culture negative	12/56 (21.4)
Gram-positive organisms only	17/44 (38.6)
Gram-negative organisms only	14/44 (31.8)
Polymicrobial infection	13/44 (29.5)
Osteomyelitis	16/60 (26.7)
No osteomyelitis	44/60 (73.3)
Isolated microorganisms§	
*Staphylococcus aureus*	16/57 (28.1)
*Pseudomonas aeruginosa*	11/57 (19.3)
*Escherichia coli*	9/57 (15.8)
*Enterococcus* spp.	7/57 (12.3)
*Klebsiella pneumoniae*	6/57 (10.5)
Other organisms	8/57 (14.0)
Total isolates	57/57 (100.0)

Data are presented as n (%). Percentages were calculated among patients with available microbiological cultures (n=56). Microbiological categories were calculated among culture-positive patients (n=44). total of 57 clinically relevant isolates were recovered from 44 culture-positive patients; because some patients had polymicrobial infections, the number of isolates exceeded the number of culture-positive patients. Osteomyelitis prevalence was calculated among all patients with DFI (n=60).

**Table 7:** Exploratory comparison of serum hBD-3 according to osteomyelitis status and microbiological category.

Group	n	hBD-3, pg/mL, M (P25, P75)	Statistic	*P*
Osteomyelitis status				
Osteomyelitis present	16	150.0 (137.0, 163.5)	*U* = 561.000	<0.001
Osteomyelitis absent	44	119.0 (97.5, 140.5)	—	—
Microbiological category				
Gram-positive organisms only	17	112.0 (100.0, 128.0)	*H* = 12.906	0.002
Gram-negative organisms only	14	121.5 (107.3, 136.5)	—	—
Polymicrobial infection	13	146.0 (134.0, 160.0)	—	—

*Post-hoc* comparisons after Bonferroni correction: polymicrobial infection vs. Gram-positive organisms only, *P* = 0.002; polymicrobial infection vs. Gram-negative organisms only, *P* = 0.035; Gram-positive organisms only vs. Gram-negative organisms only, *P* = 0.933.

hBD-3 values are expressed as M (P25, P75). The Mann-Whitney U test was used to compare patients with and without osteomyelitis. The Kruskal-Wallis H test was used to compare the three microbiological categories. The microbiological-category analysis included the 44 culture-positive patients. Because of the limited subgroup sample sizes, these comparisons were considered exploratory.

### 3.7 Comparison of serum hBD-3 and inflammatory markers according to infection severity

Among the 60 patients with DFI, 18 had mild infection, 29 had moderate infection, and 13 had severe infection. Serum hBD-3 levels were 88.5 (75.6, 101.7), 116.7 (97.2, 136.4), and 153.8 (130.6, 181.5) pg/mL in the mild, moderate, and severe groups, respectively, and differed significantly across infection-severity categories (*P* < 0.001). WBC (*P* = 0.001), CRP (*P* < 0.001), ESR (*P* < 0.001), PCT (*P* < 0.001), and NLR (*P* < 0.001) also differed significantly across the three severity groups (**Table 8**).

**Table 8:** Comparison of laboratory indicators according to infection severity.

Indicator	Mild infection (n=18)	Moderate infection (n=29)	Severe infection (n=13)	Statistic	*P* value
hBD-3 (pg/mL)	88.5 (75.6, 101.7)	116.7 (97.2, 136.4)	153.8 (130.6, 181.5)	*H* = 24.972	<0.001
WBC (×10^9^/L)	8.18 ± 1.55	9.43 ± 1.87	11.10 ± 2.23	*F* = 9.245	0.001
CRP (mg/L)	18.9 (12.4, 29.8)	43.0 (29.1, 60.7)	80.7 (59.2, 109.6)	*H* = 25.886	<0.001
ESR (mm/h)	37 (29, 48)	54 (42, 66)	73 (60, 86)	*H* = 19.892	<0.001
PCT (ng/mL)	0.11 (0.07, 0.18)	0.25 (0.15, 0.40)	0.65 (0.39, 0.98)	*H* = 22.613	<0.001
NLR	3.15 (2.49, 3.88)	4.45 (3.39, 5.40)	6.08 (4.86, 7.60)	*H* = 18.034	<0.001

### 3.8 Correlations of serum hBD-3 with infection severity and clinical indicators

Spearman correlation analysis showed that serum hBD-3 was positively correlated with IWGDF/IDSA infection grade (*r_s_* = 0.625, *P* < 0.001), Wagner grade (*r_s_* = 0.562, *P* < 0.001), CRP (*r_s_* = 0.598, *P* < 0.001), ESR (*r_s_* = 0.518, *P* < 0.001), PCT (*r_s_* = 0.489, *P* < 0.001), NLR (*r_s_* = 0.456, *P* < 0.001), ulcer area (*r_s_* = 0.429, *P* = 0.001), and length of hospital stay (*r_s_* = 0.394, *P* = 0.002) (**Table 9**; **Figure 2**).

**Table 9:** Spearman correlation analysis between serum hBD-3 and clinical indicators.

Variable	*r_s_*	*P* value
IWGDF/IDSA infection grade	0.625	<0.001
Wagner grade	0.562	<0.001
CRP	0.598	<0.001
ESR	0.518	<0.001
PCT	0.489	<0.001
NLR	0.456	<0.001
Ulcer area	0.429	0.001
Length of hospital stay	0.394	0.002

**Figure 2:**
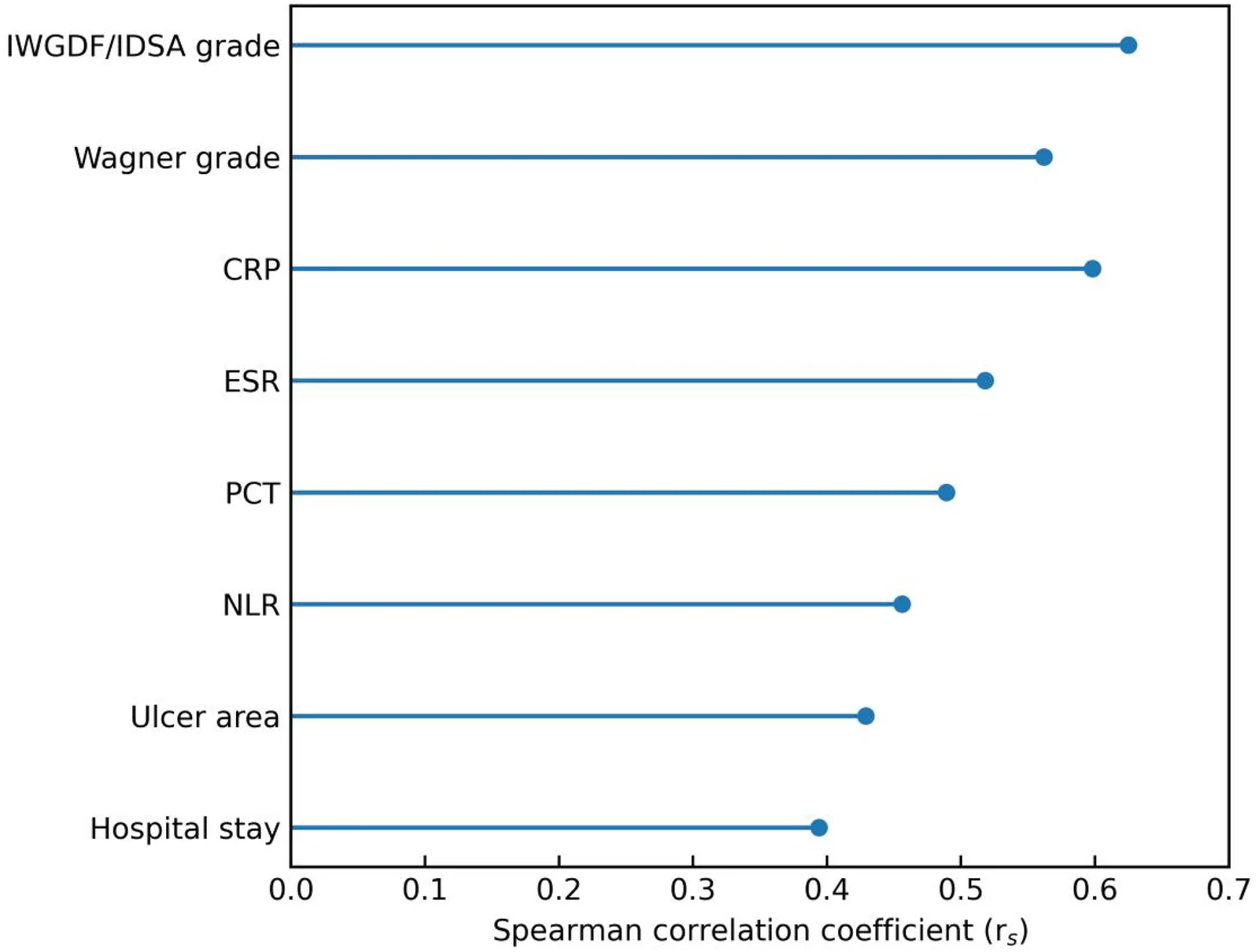
Spearman correlation coefficients between serum hBD-3 and infection severity/related clinical indicators.

### 3.9 LASSO penalized regression and multivariable analysis for prevalent DFI

Among the 90 patients with diabetic foot disease, 60 had DFI and 30 had non-infected diabetic foot disease. Univariate logistic regression showed that diabetes duration, HbA1c, albumin, hBD-3, WBC, CRP, ESR, PCT, and NLR were associated with prevalent DFI at *P* < 0.10; peripheral arterial disease was additionally included because of its clinical relevance. The 10 candidate variables were entered into LASSO penalized logistic regression. Stratified 10-fold cross-validation yielded λmin=1.796 and λ1se=4.075. At λ1se, HbA1c, albumin, hBD-3, CRP, and PCT retained non-zero coefficients, whereas diabetes duration, peripheral arterial disease, WBC, ESR, and NLR were shrunk to zero. The standardized coefficients for HbA1c, albumin, hBD-3, CRP, and PCT were 0.214, −0.328, 0.692, 0.417, and 0.286, respectively. The five retained variables were subsequently entered into multivariable logistic regression. Higher HbA1c (*P* = 0.038), hBD-3 (*P* < 0.001), CRP (*P* = 0.004), and PCT (*P* = 0.015) were associated with greater odds of prevalent DFI, whereas higher albumin was associated with lower odds of prevalent DFI (*P* = 0.007). The final combined model for prevalent DFI therefore consisted of HbA1c, albumin, hBD-3, CRP, and PCT. The corresponding regression coefficients were 0.308, −0.115, 0.351, 0.272, and 0.395, respectively, with an intercept of −1.952. The linear predictor was calculated as: LPDFI = −1.952 + 0.308×HbA1c − 0.115×Albumin + 0.351×(hBD-3/10) + 0.272×(CRP/10) + 0.395×(PCT/0.1).

The predicted probability of prevalent DFI was calculated as: PDFI = exp(LPDFI)/[1 + exp(LPDFI)]. HbA1c was entered per 1% increase, albumin per 1 g/L increase, hBD-3 per 10 pg/mL increase, CRP per 10 mg/L increase, and PCT per 0.1 ng/mL increase (**Table 10**; **Figure 3**).

**Table 10:** Univariate logistic regression, LASSO selection, and multivariable analysis for prevalent DFI.

Variable	Univariate OR (95% CI)	*P* value	LASSO coefficient	Final logistic β coefficient	Multivariable OR (95% CI)	*P* value
Diabetes duration, per 1 year	1.109 (1.011–1.217)	0.029	0.000	—	—	—
HbA1c, per 1%	1.702 (1.244–2.329)	0.001	0.214	0.308	1.361 (1.017–1.821)	0.038
Albumin, per 1 g/L	0.838 (0.770–0.912)	<0.001	−0.328	−0.115	0.891 (0.819–0.969)	0.007
Peripheral arterial disease	1.667 (0.674–4.124)	0.087	0.000	—	—	—
hBD-3, per 10 pg/mL	1.493 (1.282–1.739)	<0.001	0.692	0.351	1.420 (1.184–1.702)	<0.001
WBC, per 1×10^9^/L	1.402 (1.158–1.697)	0.001	0.000	—	—	—
CRP, per 10 mg/L	1.372 (1.187–1.585)	<0.001	0.417	0.272	1.312 (1.089–1.581)	0.004
ESR, per 10 mm/h	1.225 (1.063–1.412)	0.005	0.000	—	—	—
PCT, per 0.1 ng/mL	1.527 (1.173–1.987)	0.002	0.286	0.395	1.484 (1.081–2.037)	0.015
NLR, per 1 unit	1.276 (1.095–1.486)	0.002	0.000	—	—	—

**Figure 3:**
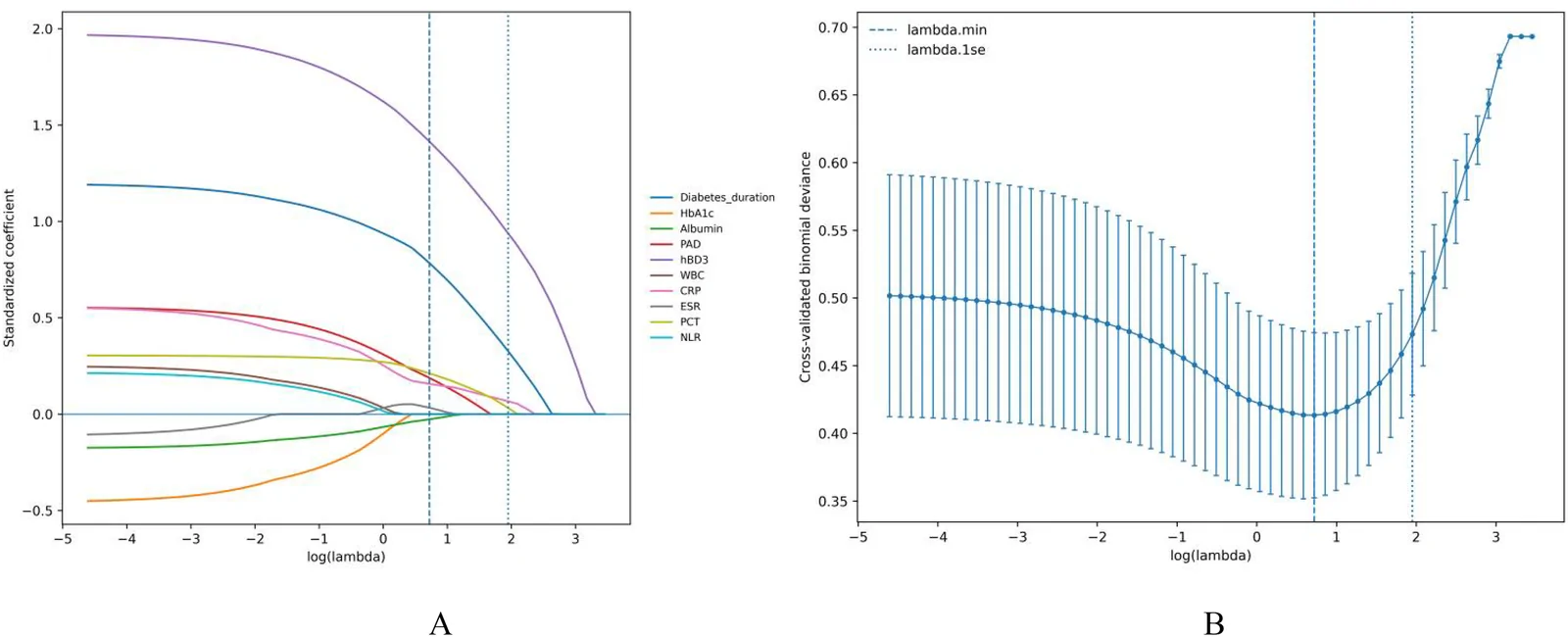
LASSO penalized regression for variables associated with prevalent diabetic foot infection. **(A)** LASSO coefficient profiles of the candidate variables. **(B)** Selection of the penalty parameter λ using stratified 10-fold cross-validation. The vertical dashed and dotted lines represent λmin and λ1se, respectively; λ1se was selected to obtain a more parsimonious model.

### 3.10 Sensitivity analysis adjusting for ulcer severity

In the sensitivity analysis addressing potential confounding by wound severity, the association between serum hBD-3 and prevalent DFI was attenuated but remained statistically significant after adjustment for ulcer duration, ulcer area, Wagner grade, and peripheral arterial disease. Each 10 pg/mL increase in serum hBD-3 was associated with higher odds of prevalent DFI (adjusted OR = 1.281, 95% CI 1.071–1.532, *P* = 0.007). Ulcer area was also associated with prevalent DFI (adjusted OR = 1.104 per 1 cm^2^ increase, 95% CI 1.016–1.199, *P* = 0.020), as was Wagner grade (adjusted OR = 1.865 per one-grade increase, 95% CI 1.184–2.936, *P* = 0.007). In contrast, ulcer duration (adjusted OR = 1.176 per 30-day increase, 95% CI 0.997–1.387, *P* = 0.054) and peripheral arterial disease (adjusted OR = 1.296, 95% CI 0.459–3.660, *P* = 0.625) were not statistically significant. All variance inflation factor values were <2.0, indicating no substantial multicollinearity. These findings suggest that the association between serum hBD-3 and prevalent DFI persisted after adjustment for selected wound-severity characteristics; however, residual confounding by unmeasured aspects of tissue injury, ischemia, or wound severity cannot be excluded (**Table 11**).

**Table 11:** Sensitivity analysis of the association between serum hBD-3 and prevalent DFI after adjustment for ulcer-severity characteristics.

Variable	*β*	Adjusted OR	95% CI	*P* value
hBD-3, per 10 pg/mL increase	0.248	1.281	1.071–1.532	0.007
Ulcer duration, per 30-d increase	0.162	1.176	0.997–1.387	0.054
Ulcer area, per 1 cm^2^ increase	0.099	1.104	1.016–1.199	0.020
Wagner grade, per 1-grade increase	0.623	1.865	1.184–2.936	0.007
Peripheral arterial disease	0.259	1.296	0.459–3.660	0.625

### 3.11 LASSO penalized regression and multivariable analysis for moderate/severe DFI

Among the 60 patients with DFI, 18 had mild infection and 42 had moderate/severe infection. The six candidate biomarkers, including hBD-3, CRP, PCT, ESR, WBC, and NLR, were entered into LASSO penalized logistic regression. Stratified 10-fold cross-validation yielded λmin=1.566 and λ1se=4.672. At λ1se, hBD-3, CRP, and PCT retained non-zero coefficients of 0.624, 0.472, and 0.288, respectively, whereas ESR, WBC, and NLR were shrunk to zero. In the subsequent multivariable logistic regression, hBD-3 (*OR* = 1.374, 95% CI 1.137–1.661, *P* = 0.001), CRP (*OR* = 1.238, 95% CI 1.048–1.463, *P* = 0.012), and PCT (*OR* = 1.443, 95% CI 1.078–1.932, *P* = 0.014) remained associated with higher odds of moderate/severe DFI. The final combined model for moderate/severe DFI consisted of hBD-3, CRP, and PCT. The regression coefficients for hBD-3, CRP, and PCT in the final multivariable logistic regression model were 0.318, 0.213, and 0.367, respectively. The intercept was −4.212. The linear predictor was calculated as: LPseverity = −4.212 + 0.318×(hBD-3/10) + 0.213×(CRP/10) + 0.367×(PCT/0.1). The predicted probability of moderate/severe DFI was calculated as: Pseverity = exp(LPseverity)/[1+exp(LPseverity)] (**Table 12**; **Figure 4**).

**Table 12:** LASSO selection and multivariable logistic regression for moderate/severe DFI.

Variable	LASSO coefficient	Final logistic β coefficient	Retained	*OR*	95% CI	*P*
hBD-3, per 10 pg/mL	0.624	0.318	Yes	1.374	1.137–1.661	0.001
CRP, per 10 mg/L	0.472	0.213	Yes	1.238	1.048–1.463	0.012
PCT, per 0.1 ng/mL	0.288	0.367	Yes	1.443	1.078–1.932	0.014
ESR, per 10 mm/h	0.000	—	No	—	—	—
WBC, per 1×10^9^/L	0.000	—	No	—	—	—
NLR, per 1 unit	0.000	—	No	—	—	—

**Figure 4:**
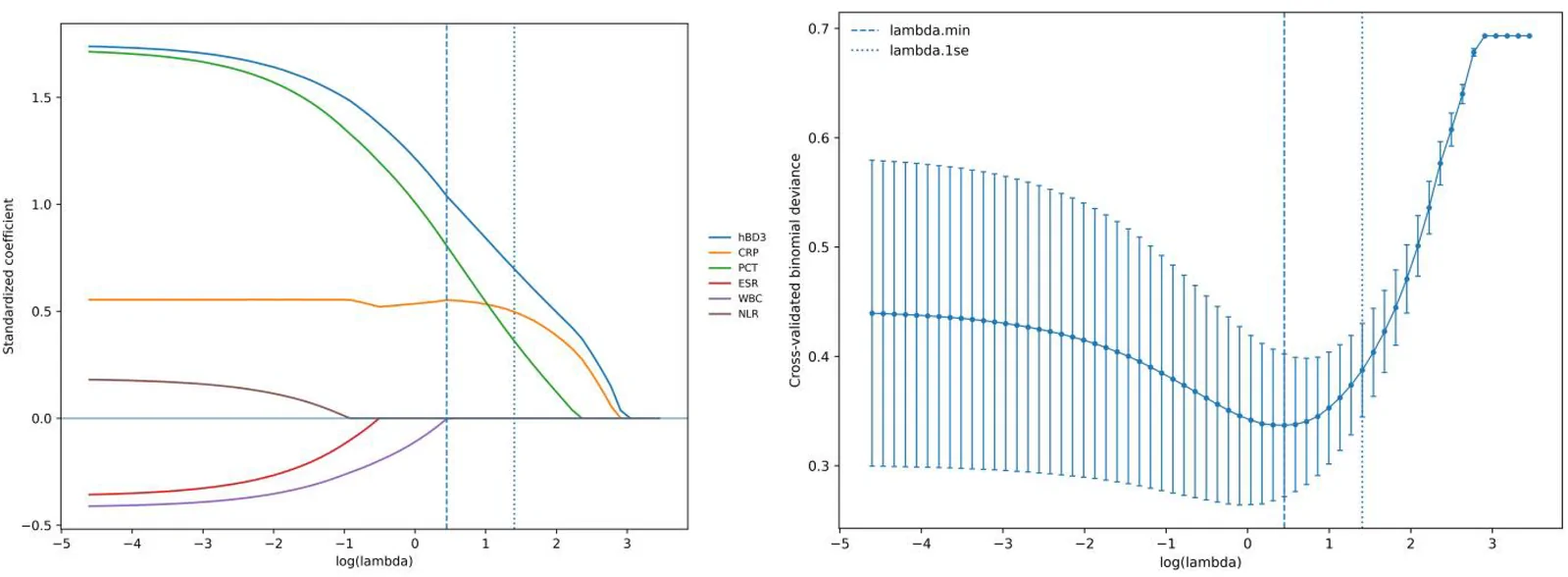
LASSO penalized regression for biomarkers associated with moderate/severe diabetic foot infection. **(A)** Coefficient profiles of the six candidate biomarkers. **(B)** Selection of the penalty parameter λ using stratified 10-fold cross-validation. λ1se was selected for the final parsimonious model.

### 3.12 Restricted cubic spline analysis

Exploratory RCS analysis using three knots at the 10th, 50th, and 90th percentiles suggested a nonlinear association between serum hBD-3 and the odds of prevalent DFI. The knots were located at 60.4, 95.7, and 152.5 pg/mL, with 95.7 pg/mL as the reference value. After adjustment for HbA1c, albumin, CRP, and PCT, the overall association remained statistically significant (*P* overall <0.001), with evidence of nonlinearity (*P* for nonlinearity = 0.021). The estimated odds generally increased with increasing hBD-3, although the 95% confidence intervals widened toward both extremes of the distribution. For CRP, knots were located at 6.6, 22.2, and 77.0 mg/L, with 22.2 mg/L as the reference value. After adjustment for HbA1c, albumin, hBD-3, and PCT, CRP showed an approximately linear positive association with prevalent DFI (*P* overall <0.001; *P* for nonlinearity = 0.284). Among the 60 patients with DFI, exploratory RCS analysis of hBD-3 for moderate/severe infection used knots at 77.4, 111.0, and 160.0 pg/mL, with 111.0 pg/mL as the reference value. After adjustment for CRP and PCT, the overall association was statistically significant (*P* overall <0.001), with evidence of nonlinearity (*P* for nonlinearity = 0.034). However, because only 18 patients had mild infection and observations were sparse at the extremes of the hBD-3 distribution, the confidence intervals widened substantially in these regions. Therefore, the apparent nonlinear pattern should be interpreted cautiously and regarded as hypothesis-generating rather than confirmatory (**Figures 5**–**7**).

**Figure 5:**
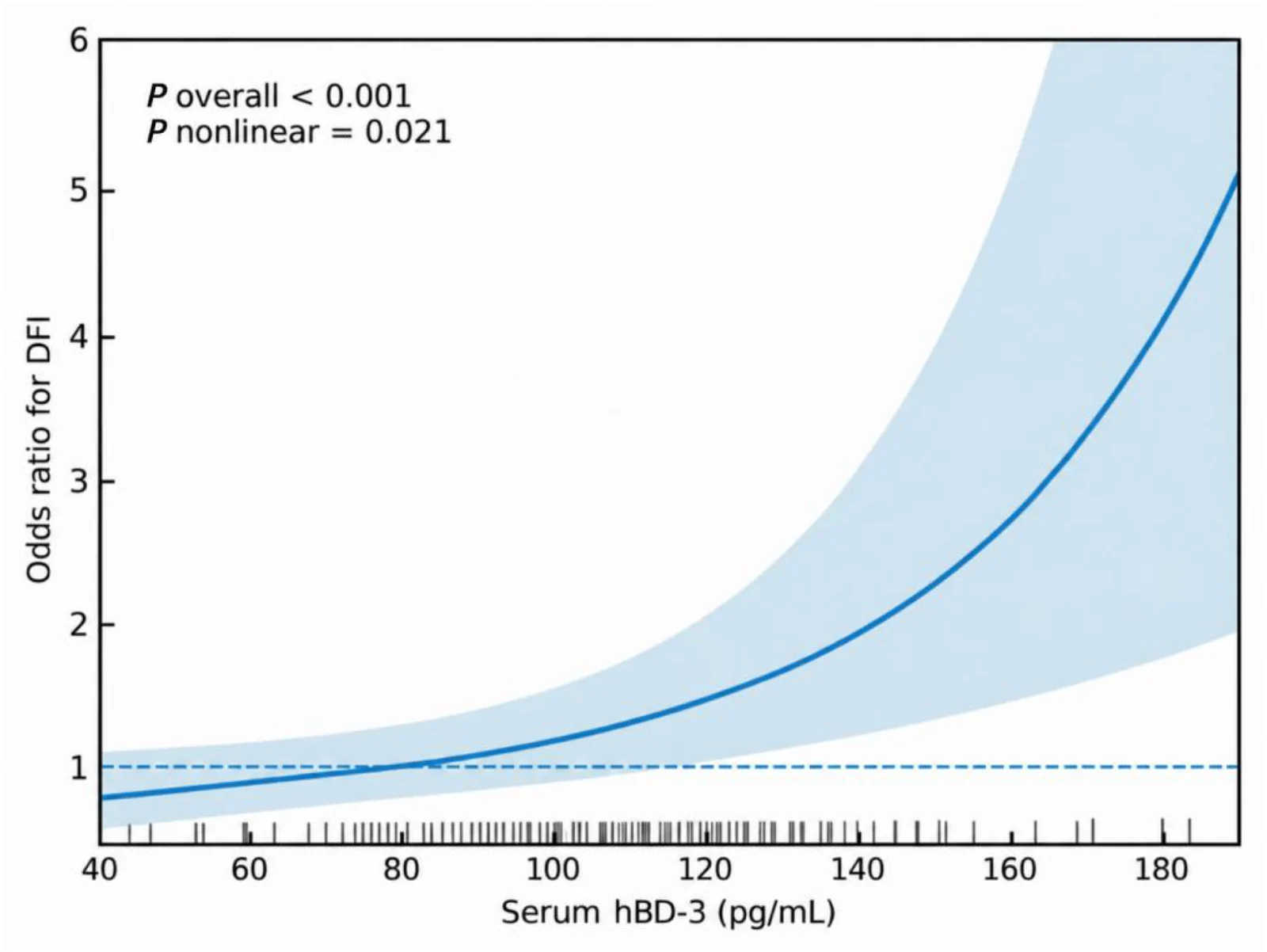
Exploratory restricted cubic spline analysis of serum hBD-3 and the odds of prevalent DFI. Three knots were placed at the 10th, 50th, and 90th percentiles (60.4, 95.7, and 152.5 pg/mL), with 95.7 pg/mL as the reference value. The model was adjusted for HbA1c, albumin, CRP, and PCT. The solid line represents the adjusted OR and the shaded area represents the 95% CI. Rug marks along the x-axis indicate the distribution of individual hBD-3 observations. *P* overall <0.001; *P* for nonlinearity = 0.021.

**Figure 6:**
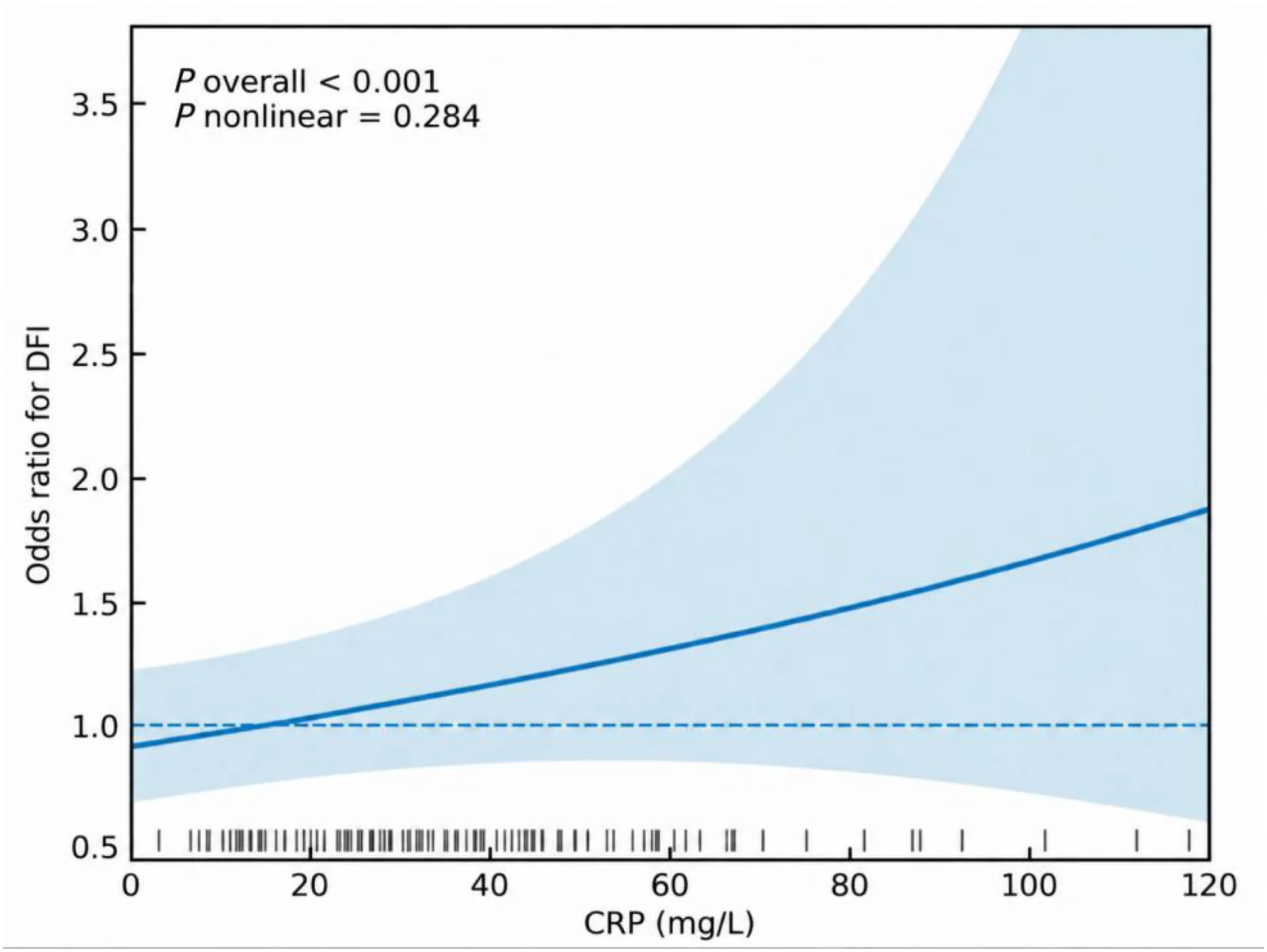
Exploratory restricted cubic spline analysis of CRP and the odds of prevalent DFI. Three knots were placed at 6.6, 22.2, and 77.0 mg/L, corresponding to the 10th, 50th, and 90th percentiles, with 22.2 mg/L as the reference value. The model was adjusted for HbA1c, albumin, hBD-3, and PCT. Rug marks indicate the distribution of CRP values. *P* overall <0.001; *P* for nonlinearity = 0.284.

**Figure 7:**
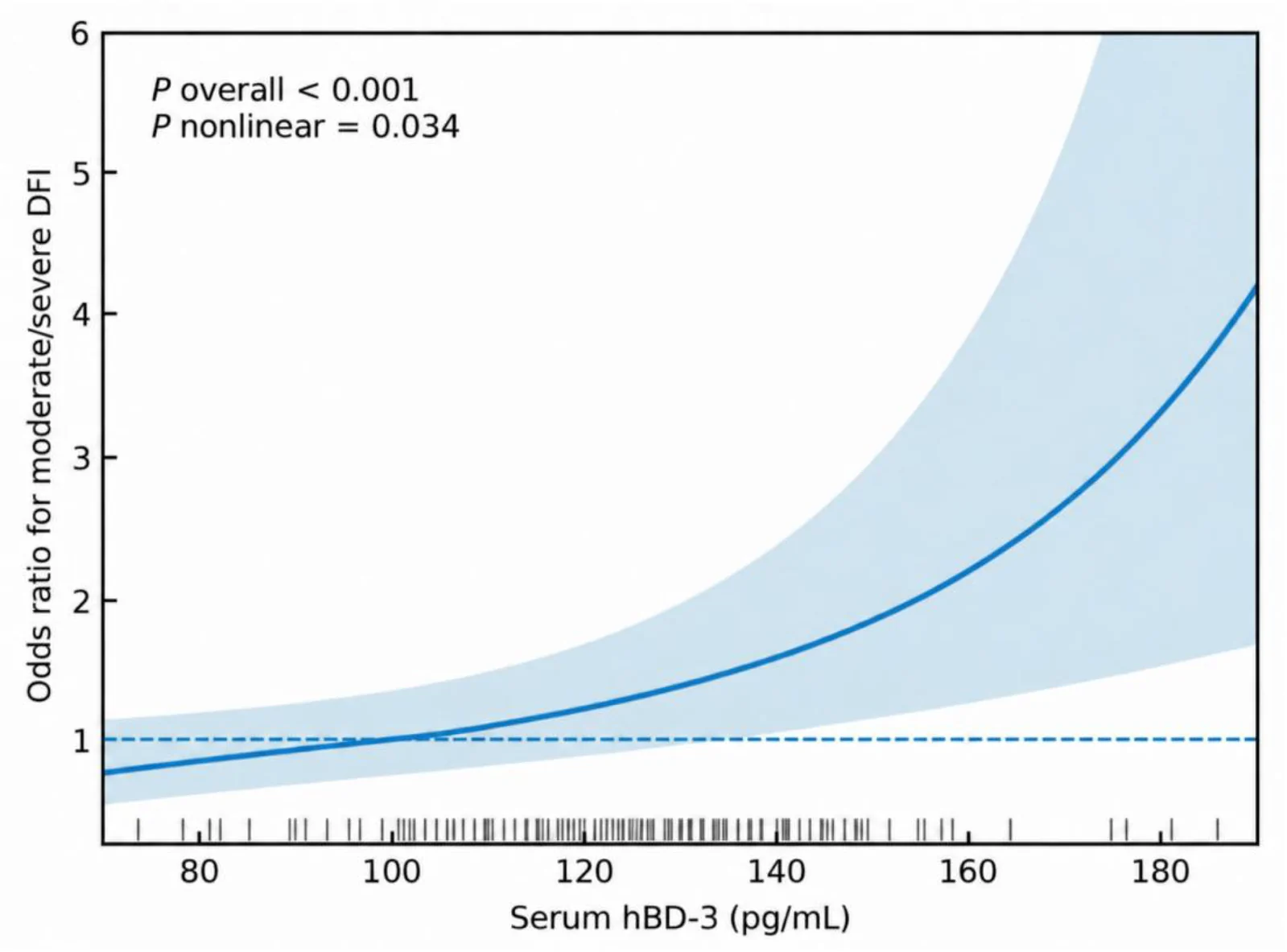
Exploratory restricted cubic spline analysis of serum hBD-3 and the odds of moderate/severe DFI. Three knots were placed at the 10th, 50th, and 90th percentiles (77.4, 111.0, and 160.0 pg/mL), with 111.0 pg/mL as the reference value. The model was adjusted for CRP and PCT. Rug marks indicate the distribution of individual hBD-3 observations. *P* overall <0.001; *P* for nonlinearity = 0.034. The widening confidence intervals at the extremes should be interpreted cautiously because of sparse observations.

### 3.13 Discriminatory performance for prevalent DFI

The combined model consisting of HbA1c, albumin, hBD-3, CRP, and PCT yielded an apparent AUC of 0.928 (95% CI 0.869–0.973), with a sensitivity of 83.3% and a specificity of 90.0%. The individual predicted probability calculated from the final logistic regression equation was used as the combined-model score. The combined model showed greater discriminatory performance than hBD-3 alone (*Z* = 2.514, *P* = 0.012), CRP (*Z* = 2.405, *P* = 0.016), PCT (*Z* = 3.041, *P* = 0.002), and NLR (*Z* = 3.715, *P* < 0.001) (**Table 13**; **Figure 8**).

**Table 13:** ROC analysis for discriminating prevalent DFI.

Indicator/model	AUC	95% CI	Optimal cutoff	Sensitivity (%)	Specificity (%)
hBD-3	0.842	0.756–0.917	87.560 pg/mL	73.3	83.3
CRP	0.824	0.721–0.913	20.930 mg/L	85.0	76.7
PCT	0.777	0.669–0.877	0.100 ng/mL	80.0	70.0
NLR	0.748	0.643–0.847	3.290	68.3	73.3
Combined model (HbA1c + albumin + hBD-3 + CRP + PCT)	0.928	0.869–0.973	0.858	83.3	90.0

**Figure 8:**
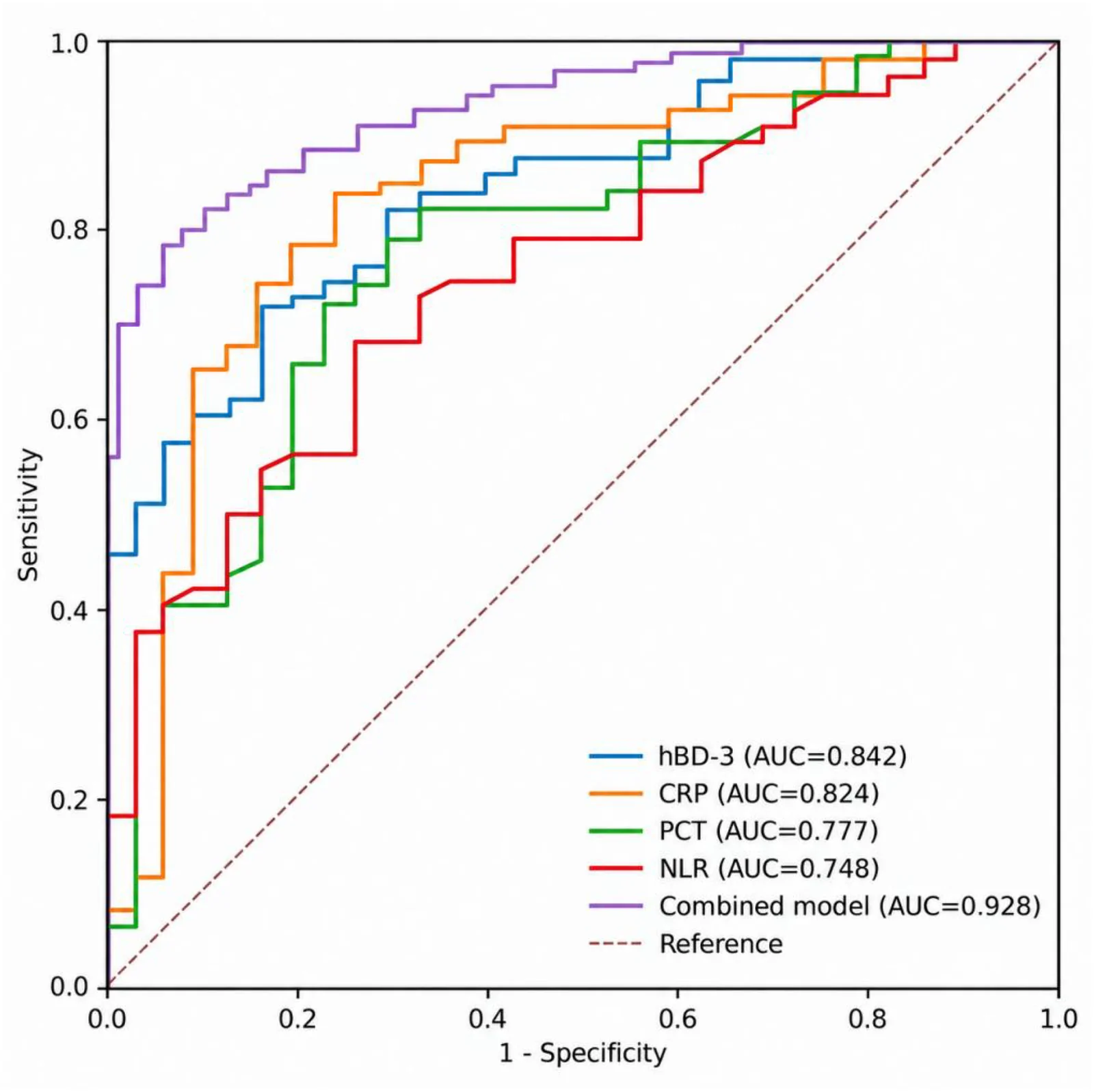
ROC curves of hBD-3, inflammatory markers, and the combined model for discriminating DFI from non-infected diabetic foot disease.

### 3.14 Discriminatory performance for moderate/severe DFI

The AUCs of hBD-3, CRP, PCT, and NLR for discriminating moderate/severe from mild DFI were 0.858 (95% CI 0.755–0.962), 0.831 (95% CI 0.720–0.941), 0.806 (95% CI 0.676–0.935), and 0.767 (95% CI 0.626–0.908), respectively.

The final LASSO-derived combined model consisted of hBD-3, CRP, and PCT. The individual predicted probability calculated from the final logistic regression equation was used as the combined-model score. The model achieved an apparent AUC of 0.935 (95% CI 0.867–0.988), with a sensitivity of 90.5% and a specificity of 88.9%. Its AUC was greater than those of hBD-3 (*Z* = 2.308, *P* = 0.021), CRP (*Z* = 2.927, *P* = 0.003), PCT (*Z* = 3.782, *P* < 0.001), and NLR (*Z* = 3.741, *P* < 0.001) (**Table 14**; **Figure 9**).

**Table 14:** ROC analysis of individual markers and the combined model for discriminating moderate/severe from mild DFI.

Indicator/model	AUC	95% CI	Optimal cutoff	Sensitivity (%)	Specificity (%)
hBD-3	0.858	0.755–0.962	123.590 pg/mL	88.1	77.8
CRP	0.831	0.720–0.941	42.970 mg/L	90.5	61.1
PCT	0.806	0.676–0.935	0.492 ng/mL	88.1	66.7
NLR	0.767	0.626–0.908	4.951	85.7	72.2
Combined model (hBD-3 + CRP + PCT)	0.935	0.867–0.988	0.714	90.5	88.9

**Figure 9:**
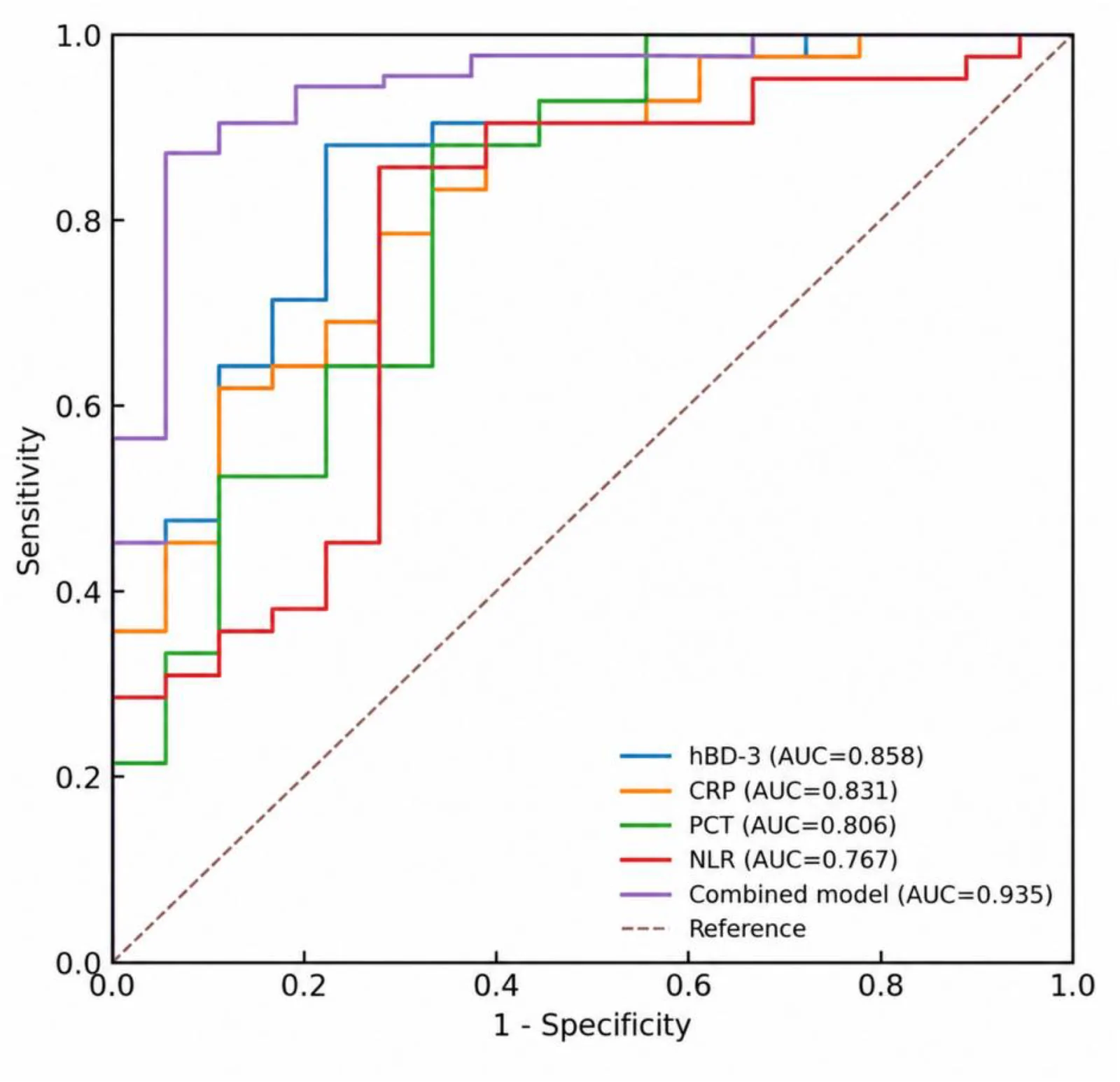
ROC curves of hBD-3, inflammatory markers, and the combined model for discriminating moderate/severe from mild DFI.

### 3.15 Internal validation of the penalized models

Internal validation was performed using 1,000 bootstrap resamples. For the prevalent DFI model, the apparent AUC was 0.928, and the optimism-corrected AUC was 0.895, corresponding to an estimated optimism of 0.033. The bootstrap-corrected calibration intercept was 0.042 and the calibration slope was 0.872, with a Brier score of 0.134. For the moderate/severe DFI model, the apparent AUC was 0.935 and the optimism-corrected AUC was 0.917, corresponding to an estimated optimism of 0.018. The bootstrap-corrected calibration intercept was 0.031 and the calibration slope was 0.901, with a Brier score of 0.122. Bootstrap-corrected calibration curves showed generally good agreement between the estimated probabilities and observed proportions within the present dataset. However, the reductions in AUC after optimism correction and calibration slopes below 1 indicated residual overfitting. Therefore, although internal validation supported the stability of the observed discriminatory performance, these models should remain exploratory until their performance is confirmed in independent external cohorts (**Table 15**; **Figures 10**, **11**).

**Table 15:** Bootstrap internal validation of the penalized models.

Model	Apparent AUC	Optimism	Optimism-corrected AUC	Calibration intercept	Calibration slope	Brier score
Prevalent DFI	0.928	0.033	0.895	0.042	0.872	0.134
Moderate/severe DFI	0.935	0.018	0.917	0.031	0.901	0.122

**Figure 10:**
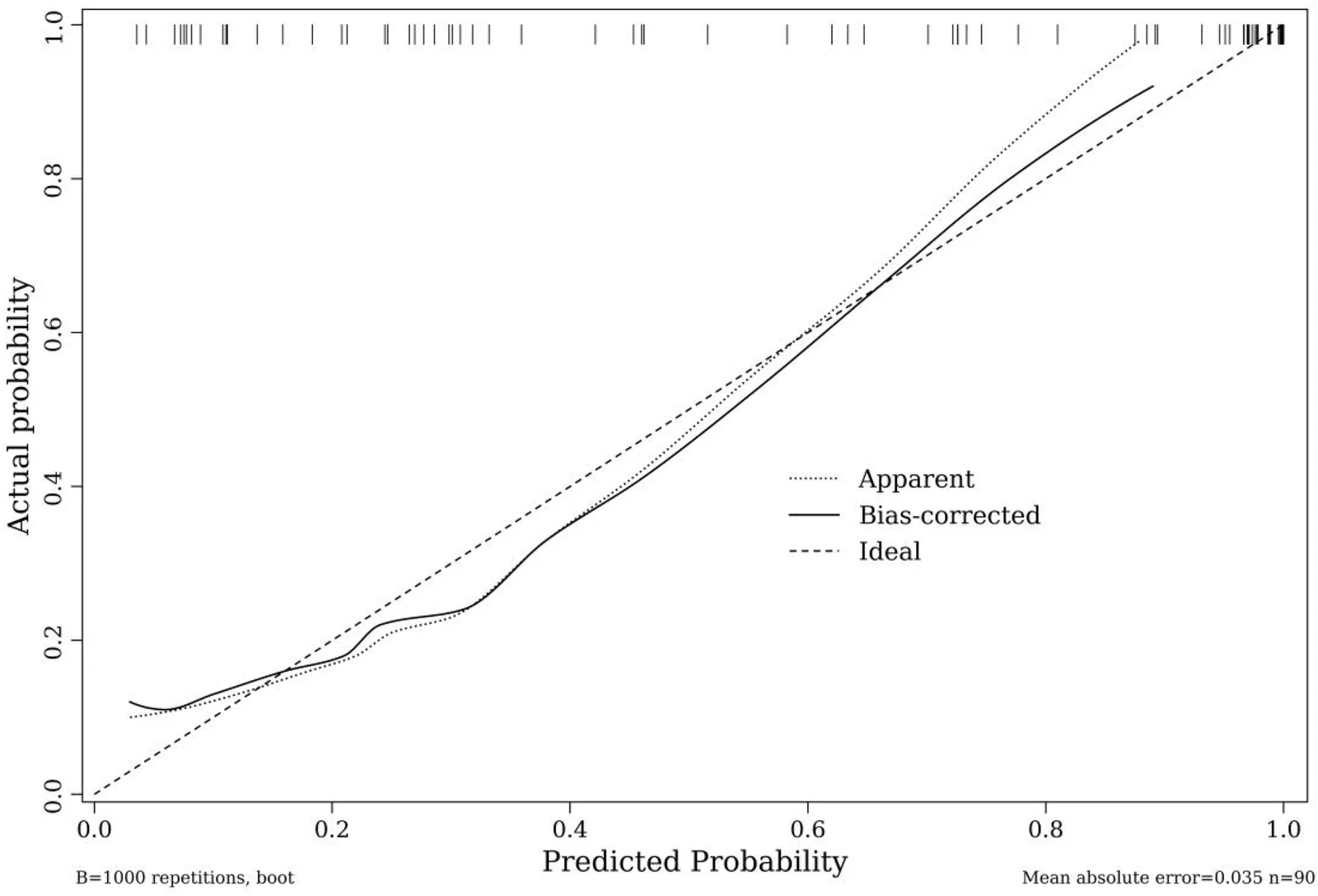
Prevalent DFI bootstrap-corrected calibration curve.

**Figure 11:**
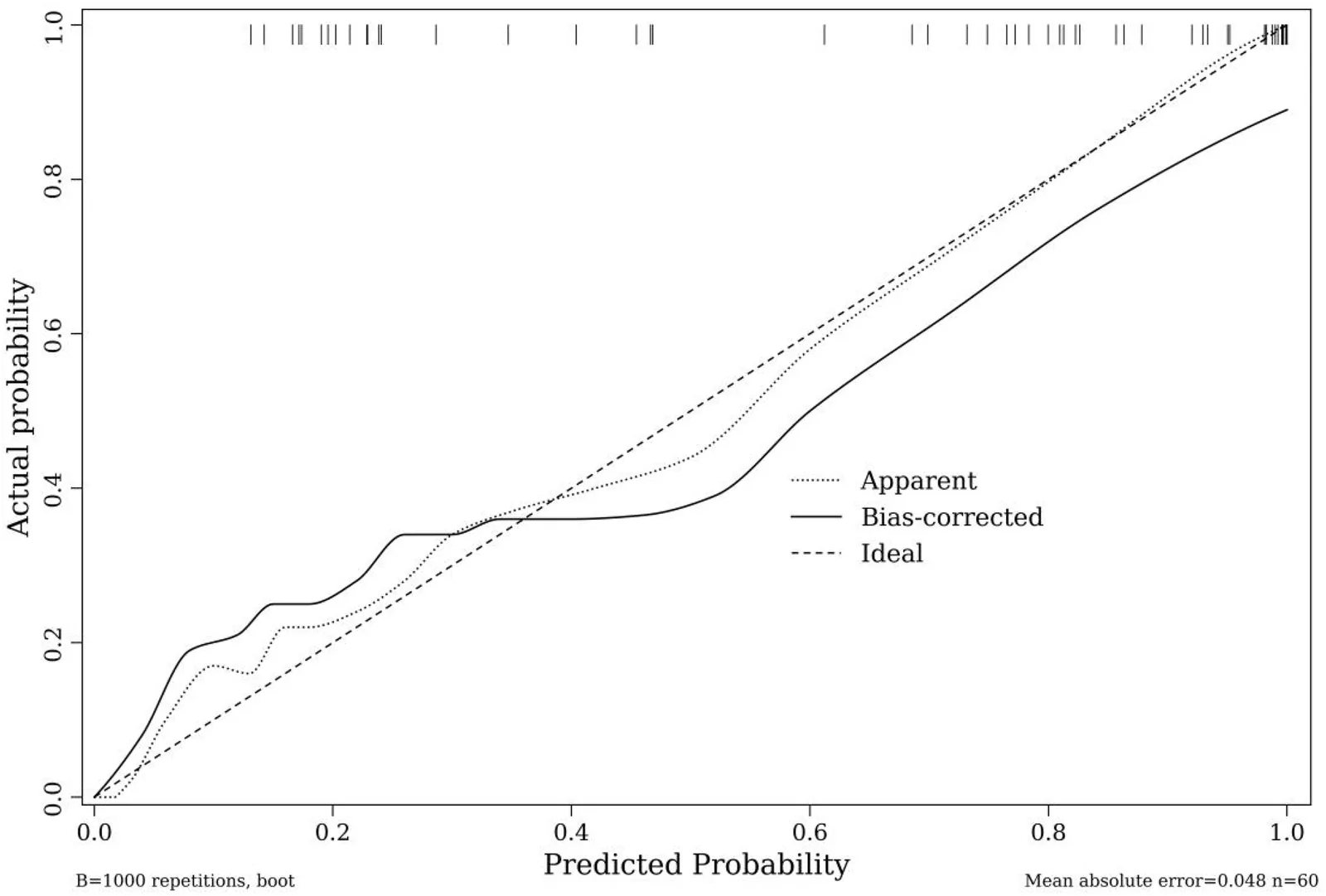
Moderate/severe DFI bootstrap-corrected calibration curve.

## 4 Discussion

Diabetic foot infection (DFI) is a clinically heterogeneous condition in which infection status and severity reflect the combined effects of microbial invasion, tissue injury, glycemic control, tissue perfusion, nutritional status, and host inflammatory responses. Therefore, circulating biomarkers should be regarded as adjuncts to established clinical diagnostic criteria rather than substitutes for clinical assessment. Previous studies have shown that PCT, CRP, NLR, and albumin may provide useful information for the assessment of diabetic foot disease, although the diagnostic performance of individual markers is variable (9–12). In the present study, serum hBD-3 increased progressively across healthy controls, patients with diabetes without foot disease, patients with non-infected diabetic foot disease, and patients with DFI, and further increased with infection severity. More importantly, the clinically relevant comparison showed significantly higher hBD-3 levels in patients with DFI than in those with non-infected diabetic foot disease. Multivariable analyses showed that higher hBD-3, CRP, PCT, and HbA1c levels and lower albumin levels were independently associated with prevalent DFI, whereas higher hBD-3, CRP, and PCT levels were associated with moderate/severe infection. After LASSO-based variable selection, the final model for prevalent DFI included HbA1c, albumin, hBD-3, CRP, and PCT, whereas the model for moderate/severe DFI included hBD-3, CRP, and PCT. The apparent AUCs were 0.928 and 0.935, respectively, and bootstrap internal validation yielded optimism-corrected AUCs of 0.895 and 0.917, with calibration slopes of 0.872 and 0.901. These results indicate that the combined models retained useful discriminatory performance after internal correction, although the reductions in AUC and calibration slopes below 1 suggest some residual overfitting. Accordingly, the present findings should be interpreted within a framework of association and diagnostic discrimination rather than prospective risk prediction, and the models should currently be regarded as exploratory rather than validated clinical decision tools.

An important issue in interpreting serum hBD-3 is whether its elevation reflects infection itself or merely greater wound severity and tissue injury. In the sensitivity analysis, the association between serum hBD-3 and prevalent DFI was attenuated but remained statistically significant after adjustment for ulcer duration, ulcer area, Wagner grade, and peripheral arterial disease (adjusted OR = 1.281 per 10 pg/mL increase, 95% CI 1.071–1.532, *P* = 0.007). This attenuation indicates that part of the hBD-3 signal overlaps with wound severity, whereas the persistent association suggests that it cannot be explained solely by ulcer size, chronicity, Wagner grade, or ischemic burden. Importantly, the non-infected comparison group consisted exclusively of patients with active diabetic foot ulcers without clinical evidence of infection, strengthening the clinical relevance of the comparison. Thus, hBD-3 is more appropriately interpreted as a complementary biomarker providing additional information on infection status rather than as an infection-specific marker. Biologically, hBD-3 is an epithelial-derived antimicrobial peptide involved in innate host defense and tissue repair, and experimental studies suggest potential roles in keratinocyte function, fibroblast migration, angiogenesis, and wound healing (13–16). Broader mechanisms involving neutrophil extracellular traps, macrophage polarization, inflammasome-related signaling, microbial burden, polymicrobial infection, antimicrobial resistance, and biofilm formation may also influence DFI-related inflammatory responses (17–26). In addition, wound healing and clinical outcomes in patients with diabetic foot disease are influenced by multiple treatment- and host-related factors, including wound management strategies, nutritional and inflammatory status, glycemic control, and other patient-specific characteristics (27–30). However, these pathways were not directly measured in the present study and should therefore be regarded only as biologically plausible hypotheses rather than mechanisms demonstrated by our data. In addition, severe DFI according to the IWGDF/IDSA classification should not be equated with sepsis. Patients meeting the criteria for severe DFI but without Sepsis-3-defined acute organ dysfunction were retained in the severe DFI group, whereas patients fulfilling the predefined Sepsis-3 criteria were excluded, thereby avoiding conflation of severe DFI with established sepsis.

Several limitations should be acknowledged. First, this was a single-center retrospective study with a limited effective sample size. Only 90 patients with diabetic foot disease were included in the prevalent DFI analysis, including 30 patients with non-infected diabetic foot disease, whereas only 60 patients with DFI were included in the infection-severity analysis, including 18 patients with mild infection. Although penalized variable selection reduced the number of predictors retained in the final models, the relatively small sample size, particularly the limited number of patients in the smaller outcome categories, continued to impose a risk of unstable regression coefficients and residual model overfitting. LASSO penalization and bootstrap internal validation were used to mitigate these concerns; however, these approaches cannot fully compensate for the limited amount of information available for model development. Accordingly, the present models should be regarded as exploratory and require validation in independent, preferably prospective multicenter cohorts. Second, serum hBD-3 was measured only once using stored samples; hBD-3 in wound tissue or exudate was not simultaneously assessed, and serial measurements before and after treatment were unavailable. Third, microbiological cultures were unavailable for some patients, and the small microbiological subgroups limited reliable evaluation of pathogen-specific hBD-3 responses. Fourth, 15 of the 60 patients with DFI had received systemic antimicrobial therapy for ≤72 h before blood sampling. Although antimicrobial exposure did not differ significantly across infection-severity groups, short-term treatment may still have influenced inflammatory biomarkers and serum hBD-3. Fifth, 14 potentially eligible individuals were excluded because of missing key clinical data, and this complete-case selection process may have introduced selection bias. Finally, ROC-derived cutoff values were developed and evaluated in the same cohort and should therefore be regarded as sample-specific exploratory thresholds rather than established clinical cutoffs. Future prospective multicenter studies should independently validate the present findings, preferentially obtain samples before antimicrobial treatment, evaluate dynamic hBD-3 changes in serum and wound-derived specimens, and develop and externally validate a standardized integrated inflammatory index combining hBD-3 with CRP, PCT, and other independently informative clinical or laboratory parameters.

## 5 Conclusion

Elevated serum hBD-3 was associated with the presence and increasing severity of DFI and provided complementary discriminatory information beyond conventional inflammatory markers such as CRP and PCT. Combined models incorporating hBD-3 with inflammatory and clinical variables showed promising performance for distinguishing DFI from non-infected diabetic foot disease and moderate/severe from mild DFI. However, because of the retrospective single-center design, limited sample size, residual risk of overfitting, and absence of independent external validation, these models should currently be regarded as exploratory rather than validated clinical decision tools. Prospective multicenter studies with independent external validation are required before clinical implementation.

## Data Availability

The raw data supporting the conclusions of this article will be made available by the authors, without undue reservation.
